# Outcome of acute epididymo-orchitis: risk factors for testicular loss

**DOI:** 10.1007/s00345-023-04500-1

**Published:** 2023-07-14

**Authors:** Sarah Marie Norton, Alex Saies, Eva Browne, Beatrice Charambra, David Silviu, Nauman Nabi, Girish Nama, Subhasis Giri, Hugh D. Flood

**Affiliations:** University Hospital Limerick, Limerick, Ireland

**Keywords:** Epididymitis, Orchitis, Epididymo-orchitis (AEO)

## Abstract

**Purpose:**

Acute epididymo-orchitis (AEO) is a common urological condition characterised by pain and swelling of the epididymis which can affect men of any age. The aetiology and to some extent the management of the patient differ between paediatric and young and older adult groups.

**Methods:**

A retrospective analysis was performed at the University Hospital Limerick from 2012 to 2016. Hospital In-Patient Enquiry (HIPE) data were obtained for all patients diagnosed with orchitis, epididymitis, epididymo-orchitis or testicular abscess over this 5-year period.

**Results:**

140 patients were identified, the age range was 0–89, median age 35.6. These were then split into 3 clinical groups, pre-pubertal (Group 1, 0–15-year-olds), sexually active young men (Group 2a, 16–35-year-olds) and men over 35 (Group 2b). Nine patients had an abscess on ultrasound investigation. There was a significant correlation between the presence of an abscess and the need for an orchidectomy (2 patients, *P* = 0.035). Two patients were reported as having an atrophic testis following AEO and both were in Group 2b.

**Conclusion:**

Overall, 7/131 (5%) patients had loss or atrophy of a testicle following an episode of AEO. Nineteen patients had further readmissions with AEO (14%).

## Introduction

Acute epididymo-orchitis (AEO) is a common urological condition characterised by pain and swelling of the epididymis with involvement of the testis to a greater or lesser degree. AEO can affect men of any age. However, the aetiology and to some extent the management of the patient differ between paediatric and young and older adult groups. Three different groups are conventionally recognised. First, the paediatric age group (< 16 years old) is distinguished from adults who are in turn subdivided into young adults (16–35 years) and older adults (> 35 years) respectively. Any difference in outcome and, in particular, in the fate of the testis between these groups has not been well studied.

### Aetiology of AEO in the paediatric population

Graumann et al. showed that of 93 paediatric patients with AEO in whom urine cultures were available only 1% showed bacteriuria [1].

This differed to a study by Tran et al. who retrospectively reviewed 220 patients and found that of the 35 patients who had cultures available, 29% grew bacteria [2]. The most common precipitating factors in these cases were dysfunctional voiding or increased physical activity. Twenty-eight underwent a voiding cystourethrography (VCUG) study of whom 6 (21%) were found to have a structural defect. It must be noted however, that Tran et al. included 20 patients from the age of 16 to 19 who were therefore outside the paediatric age group and sitting firmly in the young adult age group [2].

The main hypothesis for epididymitis in children is urinary outflow obstruction which leads to reflux of urine into the ejaculatory duct [3]. Conditions in children which can lead to the scenario of urethrovesical reflux, and ultimately epididymitis include posterior urethral valves, urethral strictures, recent instrumentation and bladder dysfunction [4–6]. A study of 44 paediatric patients with epididymitis was compared to a control group and reported a high incidence of recent or current viral infection, illustrated by significantly elevated antibody titres to pathogens in urine, nasopharynx and stool [7]. The authors concluded that epididymitis is usually an inflammatory response in the paediatric population. Further studies have found no predictive factor to be associated with a urological abnormality and recommended that investigations should be performed only after a second or recurrent episode of acute epididymo-orchitis [8, 9]. In cases of recurrent epididymitis in a paediatric population, other underlying pathologies need to be ruled out.

### Aetiology of AEO in adults

In the adult population, epididymitis often results from migration of a bacterium or virus from the urinary tract, but the main causative organisms vary however [10]. Sexually transmitted infections (STIs) are more common in younger adults. A detailed sexual history is important in all adults regardless of age in order to identify those at risk of a STI, as there will be crossover between ages with respect to the pathogens causing the infection.

### Men between 16 and 35 years old

In men between the ages of 16 and 35, the cause of AEO is most commonly a sexually transmitted pathogen. Berger et al. reported the predominant pathogens isolated from men younger than 35 were *Chlamydia trachomatis* and *Neisseria gonorrhoeae* [10].

### Men over 35

In men over 35, the infection is more commonly due to a urinary tract infection from Gram-negative enterococci [10]. Risks include a urological procedure, such as a cystoscopy and recent or current urinary catheterisation. Older men often have an obstructive voiding pattern due to benign prostatic hyperplasia as the cause of the initial urinary tract infection [11].

### Management of AEO

As previously discussed, due to the differences in aetiology and the type of organism causing the infection, different antibiotic regimens are recommended depending on patient age and likelihood of epididymitis caused by an STI.

Antibiotic treatment in a paediatric population is controversial given that urine cultures are usually negative. A study by Lau et al. reported no complications in boys with sterile urine treated with analgesia only [12]. This approach is supported by the observation that a recent viral infection may be a predisposing inflammatory factor [7]. Recurrent episodes of AEO warrant investigations to define an underlying anatomical cause.

Given that men in the 16–35-year-old age group are more likely to have AEO caused by a sexually transmitted disease, they should be treated with appropriate antibiotic cover. The European Association for Urology (EAU) and British Association for Sexual Health and HIV advise that in sexually active men at low risk of *N. gonorrhoeae*, a fluoroquinolone for 10–14 days would also be appropriate with or without doxycycline. Whilst in those at high risk of infection with *N. gonorrhoeae*, a single dose of ceftriaxone (or a fluoroquinolone for 10–14 days) with doxycycline for 10 to 14 days should be used [13, 14].

For patients over 35, given that the more likely causative organism is due to Gram-negative enterococci, the antibiotic management is different from their younger counterparts. As men get older, the element of urinary obstruction increases, increasing their risk of Gram-negative enterococci causing an episode of AEO. The European Association of Urology (EAU) and British Association for Sexual Health and HIV recommend that a fluoroquinolone for 10 to 14 days should be given to treat Enterobacteriaceae infection in these men [14].

Of course some crossover of causative organism will occur between younger and older adult groups so that a detailed sexual and lower urinary tract symptom history is necessary in order to tailor treatment for each patient.

### Testis outcomes

The usual course of AEO is resolution following appropriate antibiotic/anti-inflammatory treatment. Less commonly, abscess formation can occur and even more rarely, infarction which may require orchidectomy or result in testicular atrophy. It is thought that testicular ischaemia due to AEO results from exudate production and oedema leading to compression of epididymal and testicular vessels in the space confined by the unyielding tunica albuginea, ultimately causing a compartment-like syndrome. Figueroa et al. described a tunica albuginea fasciotomy in order to decrease the compartment pressure with a graft of tunica vaginalis then used for closure [15].

Desai et al. in a prospective study in 1986 of 33 patients in the age range 15–87 yr, reported testicular complications in 39% following an episode of AEO including, infarction, late atrophy, suppurative necrosis and abscess formation, with a total of 5 patients ultimately requiring an orchidectomy [16].

Whilst rare, a number of cases have been described in the literature of testicular loss due to epididymo-orchitis despite appropriate antibiotic treatment. The majority of cases are reported in adults. Alharbi et al. reported a case with abscess formation following apparently adequate treatment, which subsequently deteriorated and exploration revealed an ischaemic testicle [17]. Similarly, Fehily et al. reported 2 cases of testicular ischaemia in adults, each of whom had predisposing factors (HIV and recurrent AEO) [18].

Adorisio et al. reported the case of an 18-month-old boy with epididymitis, who was found to have a segmental haemorrhagic infarction of the testis [19].

Our aim was to further examine testicular outcomes following AEO in each age grouping and determine the risk factors for testicular loss [20, 21].

## Methods

A retrospective analysis was performed in the University Hospital Limerick from 2012 to 2016. Hospital In-Patient Enquiry (HIPE) data was obtained for all patients diagnosed with orchitis, epididymitis, epididymo-orchitis or testicular abscess over this 5-year period.

**Fig. 1:**
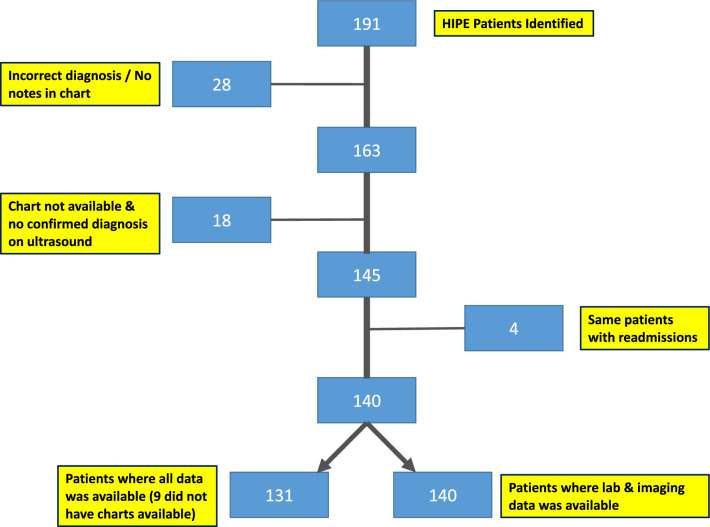
Flow Diagram for inclusion and exclusion from retrospective analysis

Included were 191 males aged 0–89 years. Of these, 28 were ruled out of the study due to an incorrect diagnosis of AEO on HIPE. A further 18 patients were excluded due to charts being unavailable and with no confirmed diagnosis of AEO on ultrasound. Of the remaining 145 patients, 4 were identified as being the same person recorded multiple times due to re-admission with the same diagnosis. Nine patient charts were unavailable, but these were included in the laboratory and ultrasound analysis as they had ultrasound reports confirming the diagnosis. (See Fig. 1).

Of the 140 patients identified, the age range was 0–89, median age 35.6. These were then split into 3 clinical groups, pre-pubertal (Group 1, 0–15-year-olds), sexually active young men (Group 2a, 16–35-year-olds) and men over 35 (Group 2b). All underwent radiological examination and laboratory blood/urine analysis. In 131 patients, charts were available for review. These were further analysed in terms of clinical presentation, review of antibiotic choices and clinical management as well as surgical intervention, length of stay and gonadal outcome between the patient groups.

### Statistical methods

The chi-square test for association was used for all variables except for temperature in which the Fisher’s exact test was used.

## Results

140 patients were divided into the three clinical groups as follows: Pre-pubertal/Peri-pubertal (0–15 years old), sexually active young men (16–35 years old) and men over 35 years old. These patients were all included in radiological and laboratory blood/urine analysis. Nine patients were excluded from chart review as charts were unavailable. The remaining 131 patients were included in clinical presentation analysis, review of antibiotic choices and patient management as well as surgical intervention, length of stay and gonadal outcome. (See Table 1 for patient breakdown per group).

**Table 1:** Clinical groups

	Group 1	Group 2a	Group 2b	Total
Age range	0–15	16–35	> 35	
Radiology & Lab analysis only available	2	1	6	9
Chart review available	41	28	62	131
Total No. of patients	43 (31%)	28 (20%)	69 (49%)	140

### Clinical presentation

Local symptoms only, such as erythema, swelling and tenderness, were more prevalent in younger age groups. One-third of Group 2b had both urinary and local symptoms at presentation and nearly 20% had these symptoms ongoing for over 1 week prior to hospital attendance (*P* = 0.003). (See Table 2).

**Table 2:** Clinical presentation

Age range (*n*)	0–15 (41)	16–35 (28)	> 35 (62)
*Symptoms*			
Local only	38 (92.7%)	24 (85.7%)	39 (62.9%)
Urinary only	0	0	2 (3.2%)
Both	3 (7.3%)	4 (14.3%)	21 (33.9%)
*Duration of symptoms*			
Not recorded	0	0	1 (2.1%)
1–3 days	27 (65.8%)	19 (67.8%)	26 (42%)
4–7 days	12 (29.3%)	8 (28.6%)	23 (37%)
7–14 days	2 (4.9%)	0	6 (9.7%)
> 2 weeks	0	1 (3.6%)	6 (9.7%)
*Prior healthcare visits*			
Not recorded	2 (4.9%)	2 (7.1%)	2 (3.2%)
0	17 (41.5%)	11 (39.3%)	27 (43.5%)
1	21 (51.2%)	12 (42.9%)	32 (51.6%)
2	0	2 (7.1%)	1 (1.6%)
> 2	1 (2.4%)	1 (3.6%)	1 (1.6%)
*Antibiotic treatment prior to hospital attendance*			
Not recorded	9 (22%)	5 (17.9%)	18 (29%)
No	27 (65.8%)	14 (50%)	28 (45.2%)
Yes	5 (12.2%)	9 (32.1%)	16 (25.8%)
*Potential risk factors for AEO (Some had* > *1)*			
Not recorded	3 (7.3%)	2 (7.1%)	2 (3.2%)
None	27 (65.8%)	14 (50%)	24 (38.7%)
Diabetes	0	1 (3.6%)	7 (11.3%)
Smoking	0	2 (7.1%)	10 (16.1%)
Steroids	0	0	2 (3.2%)
Underlying urinary issue	7 (17%)	7 (25%)	20 (32.3%)
Prior episode	3 (7.3%)	1 (3.6%)	3 (4.8%)
Unprotected sex	0	3 (10.7%)	0

There was no statistically significant correlation found between symptoms, prior healthcare visits, antibiotic treatment in the community and abscess formation or orchidectomy (chi-square test). A borderline association was found between patients with a shorter duration of symptoms and the occurrence of an abscess (*P* = 0.051).

### Risk factors for poorer outcomes: (See Table 2)

Presumptive risk factors for poorer outcomes after AEO were common in older patients. However, surprisingly, there was no significant correlation between diabetes mellitus, smoking, prior episodes or steroids and the occurrence of an abscess, orchidectomy or need for further treatment or re-admission. Patients with an underlying known urinary problem were found to be significantly more likely to develop an abscess compared to those without any known urinary issue (chi-square test, *P* = 0.022). However, there was no such correlation with the need for orchidectomy. All patients who admitted to unprotected intercourse were in the 16–35 age group (3 patients, *P* = 0.002, chi-square test) and those who had this risk factor were more likely to develop an abscess (2 patients, *P* = 0.018, chi-square test). Again however there was no correlation with the requirement for an orchidectomy Tables 3 and 4.

**Table 3:** Type of antibiotic treatment during admission

Antibiotic	Group 1	Group 2a	Group 2b
Not recorded (or unknown)	5	2	3
Co-amoxiclav	31	0	5
Ciprofloxacin	0	10	9
Ceftriaxone	0	1	3
Azithromycin /Doxycycline	0	2	1
Gentamicin	1	3	2
Other	0	0	6
Co-amox + Gent	3	5	15
Cipro + Gent	0	1	9
Gent + Other	1	2	1
3 Abx	0	0	8
Azaithromycin/doxycycline	0	2	0

**Table 4:** Total Duration of antibiotic treatment (Inpatient & Outpatient)

Days	Group 1	Group 2a	Group 2b
Not recorded	3	3	5
< 7	5	2	4
7–14	30	16	29
15–21	3	5	17
> 21	0	2	7
Total	41	28	62

### Antibiotic treatment

Seventy (54%) patients had antibiotics changed on admission. Of the 5 patients who underwent orchidectomy, 3 were treated with a combination of augmentin and gentamicin with or without additional antibiotics. Those who required antibiotic treatment beyond 2 weeks were more likely to be readmitted or to need further antibiotic treatment after later review in the outpatient clinic (*P* = 0.009, chi-square test).

### Mid-stream urine (MSU)

MSU was not done in 34 (24%) patients. Almost half (16) of these were in the 0–15 years group. Of the MSUs performed, 79/106 (73%) had no specific growth. *E. coli* was the predominant organism identified in 22/27 (81%) positive cultures and was cultured in 22/106 (21%) patients overall. Six of twenty-seven (22%) patients had evidence of antibiotic resistance. There was no significant correlation between negative outcomes and positive culture (chi-square test).

### Temperature

Of the 129 patients where temperature record was available 106 (82%) were afebrile during admission. Twenty-three of the 129 (18%) patients had temperature spikes, of whom 6/23(26%) were in Group 1, 3/26 (12%) were in Group 2a and 14/23 (60%) were in Group 2b. There was no correlation between an elevated temperature and the requirement for an orchidectomy (Fisher’s exact test *P* = 0.562) or re-admission (Fisher’s exact test *P* = 0.373).

### Infection and inflammatory blood markers

**Fig. 2:**
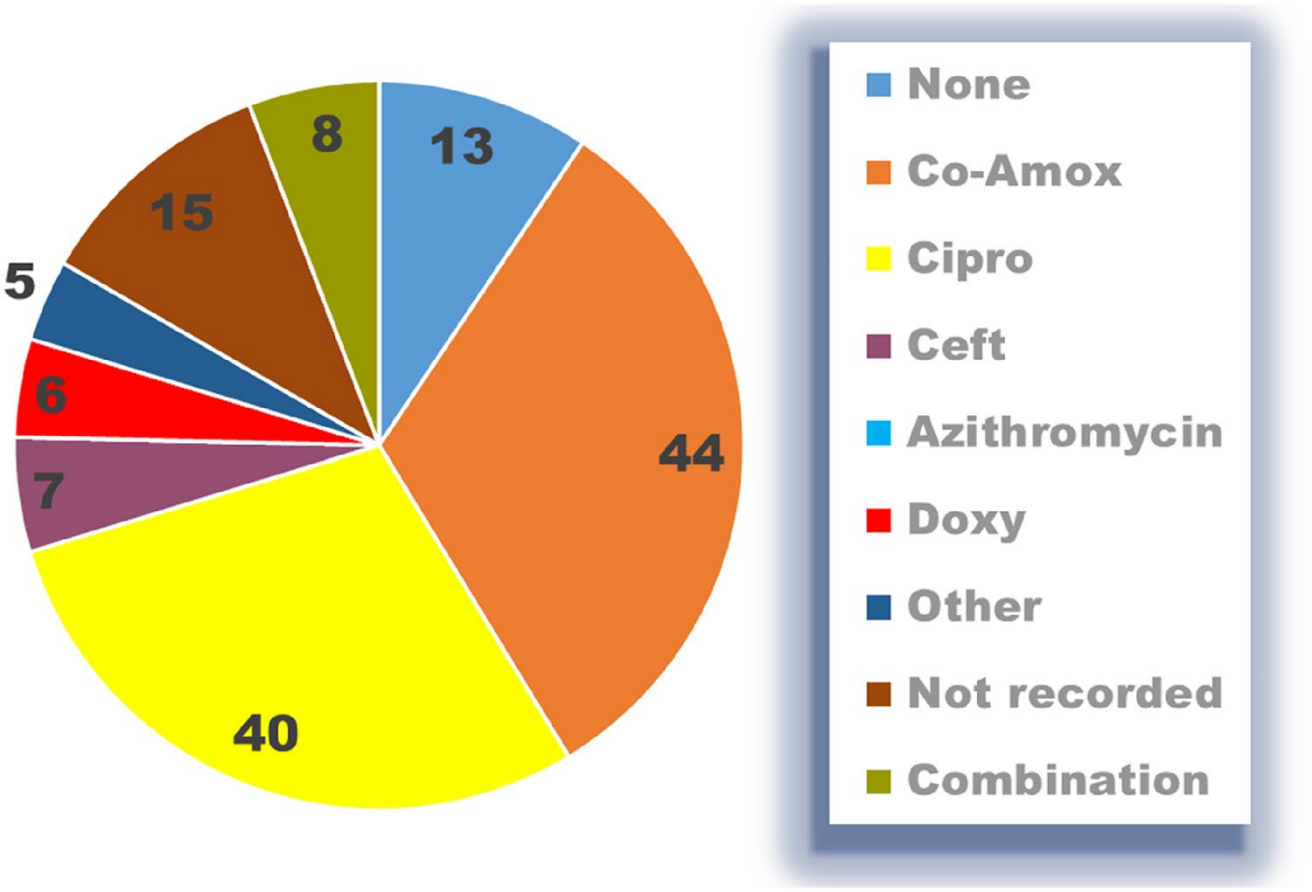
Shows the patients antibiotic treatment on discharge

An elevated white cell count (Wcc > 11) was found in 75/140 (54%) patients. Of those, the count was recorded as > 20 in 19/75 (25%) patients, none of whom were in Group 1. C-Reactive Protein (CRP) was recorded in 65/140 (46%) patients, and was < 5 in 22/65 (34%) patients where it was measured. CRP was > 50 in 34/65 (52%) patients (Fig. 2).

### Abscess and orchidectomy

**Fig. 3:**
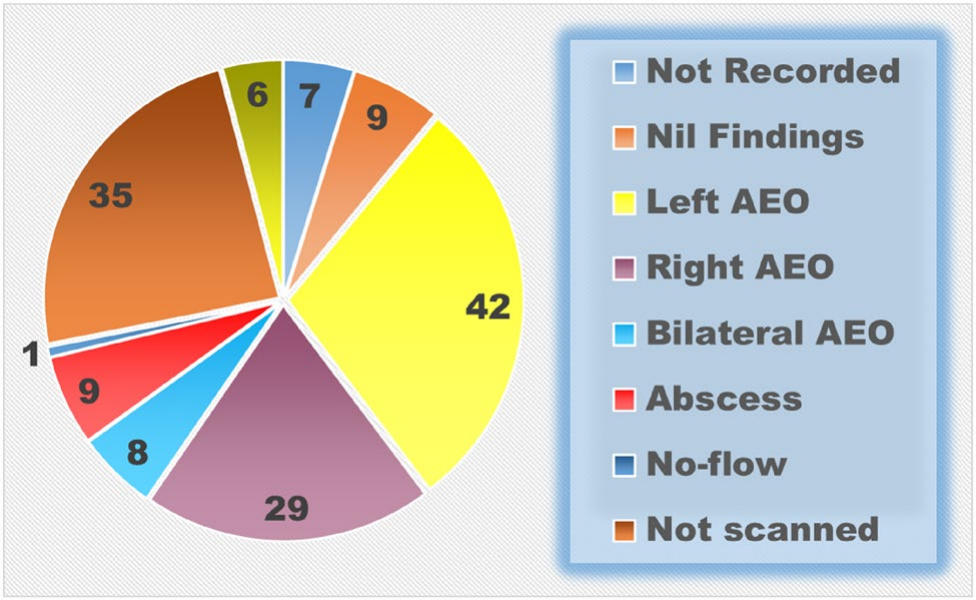
Ultrasound findings (%)

Of the 139 patients that underwent an ultrasound investigation, 9 (6%) had an abscess visualised. 1/43 (2.3%) were in Group 1, 3/28 (10.7%) in Group 2a and 5/69 (7.2%, all of whom were > 50) in Group 2b (See Fig. 3).

Five patients in total underwent orchidectomy (4%) and 4 were in Group 2b. Two patients who underwent orchidectomy had abscess formation identified on ultrasound. There was a significant correlation between the presence of an abscess and the need for an orchidectomy (2 patients, *P* = 0.035). Two patients were reported as having an atrophic testis following AEO and both were in Group 2b. Overall, 7/131 (5%) patients had loss or atrophy of a testicle.

### Further interventions/readmissions

Five older patients underwent other surgical procedures as an outpatient following their AEO which had previously contributed to the development of their AEO. One patient developed acute delirium. Ninety-seven (67%) patients required no further follow-up. Nineteen patients had further readmissions with AEO (14%).

## Discussion

Acute epididymoorchitis is a relatively common condition affecting between 25 and 65 men per 10,000 per year [21]. The aetiology and to some extent the management of the patient differ between paediatric and young and older adult groups. Several case reports of testicular loss following acute epididymoorchitis have been reported, but no large prospective study exists. In a prospective study from 35 years ago, Desai et al. reported complications of 39% including testicular infarction, atrophy and abscess formation with 15% (5 patients) ultimately requiring an orchidectomy [16]. In our study, the rates of loss of testes was relatively high in comparison to literature at 5% (7/131), 2 had testicular atrophy on follow-up and 5 underwent an orchidectomy.

### AEO in group 1 (< 16)

As previously described, AEO in the paediatric population is usually an inflammatory response. Our study shows the majority of paediatric patients tended to present within 3 days of onset of symptoms and have only localised symptoms. Three patients (7%) had recurrent episodes of AEO and 17% were found to have an underlying urinary issue. Other studies have found no predictive factor to be associated with a urological abnormality and recommended that investigations should be performed only after a second or recurrent episode of acute epididymo-orchitis [8, 9].

Half of our paediatric patients did not have any MSU sent and none had significantly raised inflammatory markers. However, 26% were found to be febrile and 1 patient had an abscess formation on ultrasound.

### AEO in Group 2a (16–35)

AEO has been reported as most commonly being caused by a sexually transmitted pathogen. In our cohort, only 10% (3 patients) in this age group reported having unprotected intercourse prior to the development of AEO. A total of 25% were found to have an underlying urinary issue. However, there was no correlation with the requirement for an orchidectomy.

### AEO in Group 2b (> 35)

In men over 35, the infection is more commonly due to a urinary tract infection from Gram-negative enterococci [10]. Often, there is an obstructive element due to benign prostatic hyperplasia as the cause of the initial urinary tract infection [11]. In our study, men over the age of 35 (Group 2b) are more likely to have both local and urinary symptoms and are twice as likely to be febrile and have more numbers of white blood cells. This study showed that this age group accounted for 4 of 5 orchidectomies related to acute epididymoorchitis (80%). These men seek medical help after a longer time period and have a greater frequency of presumptive contributing risk factors associated with acute epididymoorchitis. However, surprisingly, there was no significant correlation between diabetes mellitus, smoking, prior episodes or steroids and the occurrence of an abscess, orchidectomy or the need for further treatment or re-admission. Patients with an underlying known urinary problem were found to be significantly more likely to develop an abscess compared to those without any known urinary issue. *E. coli* was the predominant organism grown from MSU but, there was no significant correlation between negative outcomes and positive culture. Five older patients underwent other surgical procedures as an outpatient following their AEO which had previously contributed to the development of their AEO. Of the 9 patients who had an abscess identified on an ultrasound, 5 were > 50, There was a significant correlation between the presence of an abscess and the need for an orchidectomy (2 patients, *P* = 0.035).

### AEO management

Only 1 in 3 patients were treated as per EAU antibiotic guidelines within the community, prior to hospital admission. Fifty-three % of sexually active men received antibiotics as per EAU guidelines under the care of the urological team. 43% of all men > 16 received dual/triple antibiotic treatment whilst as an inpatient. This may be due to the severity of the condition, but there was no evidence that this was driven by microbiological input or culture growth. Patients who received a longer duration of antibiotics (> 2 weeks) were more likely to require further antibiotic treatment or re-admission, likely related to the severity of the condition. Twenty-four per cent had no MSU sample sent. Of those sent, 73% had no growth. *E. coli* was the predominant bacterium grown (21%). Twenty per cent of those with positive MSU samples had antibiotic resistance (6 patients). There was no correlation between positive MSU growth and negative patient outcomes.

### Testes outcome

As previously discussed, it is thought that testicular ischaemia due to AEO results from exudate production and oedema leading to compression of epididymal and testicular vessels in the space confined by the unyielding tunica albuginea, ultimately causing a compartment-like syndrome. In our study, 9 patients had abscesses related to their AEO (6%), 5 underwent orchidectomies and 2 had testicular atrophy. There was a clinically significant correlation between abscess formation and orchidectomy. However, surprisingly, there was no significant correlation between diabetes mellitus, smoking, prior episodes or steroids and the loss of the testicle.

### Study limitations

This was a retrospective analysis of patients diagnosed with acute epididymoorchitis.

## Conclusion

Our study has shown a significant rate of testicular loss following acute epididymoorchitis of 5% (7/131). Men over the age of 35, were at an increased risk of requiring an orchidectomy, accounting for 4 of 5 orchidectomies related to acute epididymoorchitis (80%). These men seek medical help after a longer time period and have a greater frequency of presumptive contributing risk factors associated with acute epididymoorchitis. However, this study did not demonstrate any correlation between testicular loss and relevant medical comorbidities, such as diabetes mellitus, smoking, recurrent epididymoorchitis or steroids. There was a significant correlation between the presence of an abscess and the need for an orchidectomy.
